# Identification of Alternatively Translated Tetherin Isoforms with Differing Antiviral and Signaling Activities

**DOI:** 10.1371/journal.ppat.1002931

**Published:** 2012-09-27

**Authors:** Luis J. Cocka, Paul Bates

**Affiliations:** Department of Microbiology, Perelman School of Medicine at the University of Pennsylvania, Philadelphia, Pennsylvania, United States of America; Vanderbilt University School of Medicine, United States of America

## Abstract

Tetherin (BST-2/CD317/HM1.24) is an IFN induced transmembrane protein that restricts release of a broad range of enveloped viruses. Important features required for Tetherin activity and regulation reside within the cytoplasmic domain. Here we demonstrate that two isoforms, derived by alternative translation initiation from highly conserved methionine residues in the cytoplasmic domain, are produced in both cultured human cell lines and primary cells. These two isoforms have distinct biological properties. The short isoform (s-Tetherin), which lacks 12 residues present in the long isoform (l-Tetherin), is significantly more resistant to HIV-1 Vpu-mediated downregulation and consequently more effectively restricts HIV-1 viral budding in the presence of Vpu. s-Tetherin Vpu resistance can be accounted for by the loss of serine-threonine and tyrosine motifs present in the long isoform. By contrast, the l-Tetherin isoform was found to be an activator of nuclear factor-kappa B (NF-κB) signaling whereas s-Tetherin does not activate NF-κB. Activation of NF-κB requires a tyrosine-based motif found within the cytoplasmic tail of the longer species and may entail formation of l-Tetherin homodimers since co-expression of s-Tetherin impairs the ability of the longer isoform to activate NF-κB. These results demonstrate a novel mechanism for control of Tetherin antiviral and signaling function and provide insight into Tetherin function both in the presence and absence of infection.

## Author Summary

Regulation of innate immunity is critical to maintain a balance between control of a perceived threat and immunopathology. The interferon induced cellular factor Tetherin has been shown to restrict budding of a broad range of enveloped viruses including the human immunodeficiency virus. Though Tetherin appears to be a bona fide viral restriction factor, additional cellular functions have been observed including an involvement in actin cytoskeleton organization in polarized cells, regulating interferon secretion and signaling through nuclear factor-kappa B (NF-κB). Our studies present a mechanism by which Tetherin function is regulated at the translational level through the production of alternatively translated isoforms. The short isoform of Tetherin was observed to be significantly more resistant to HIV-1 Vpu. In contrast, the longer isoform can induce NF-κB activity, a function lacking in the short isoform. Critical NF-κB signaling residues include a dual tyrosine motif, which is only present in the long isoform. Identification of these isoforms helps to illuminate how Tetherin functions, not only as a restriction factor, but also as a signaling molecule. These data highlight a previously unappreciated level of regulation and furthers our understanding of additional Tetherin functions.

## Introduction

Tetherin (BST-2/CD317/HM1.24) is an interferon (IFN) induced, type II transmembrane glycoprotein which has been shown to function as an intrinsic antiviral factor that restricts release of a broad range of enveloped viruses, including members of the arenavirus [1], [2], filovirus [2], [3], gamma-herpesvirus [4], [5], paramyxovirus [1], retrovirus [6]–[8] and rhabdovirus [9], [10] families. Selective pressure from Tetherin on the aforementioned families of viruses is evident because many of these Tetherin sensitive viruses encode proteins that counter Tetherin function. To date, several virally encoded Tetherin antagonists have been identified, a few of which function through different mechanisms. These include HIV-1 Vpu [11], [12], KSHV K5 [4], SIV Nef [13], HIV-2 and SIV Env [14], [15], and ebolavirus GP [3].

Tetherin has an unusual topology that has been shown to be critical for function as a restriction factor. Tetherin is a lipid raft resident protein anchored to the cellular membrane by a N-terminal transmembrane domain and a C-terminal GPI anchor [16]. Localization to sites of enveloped virus budding and anchoring at both ends is needed for Tetherin function as a cellular restriction factor through a direct “tethering” mechanism which bridges the viral and host membranes [17]. The N-terminus also includes a cytoplasmic tail, which contains a tyrosine-based motif that mediates trafficking between lipid rafts at the plasma membrane and intracellular compartments, including the trans-Golgi Network (TGN), via adaptor complexes involved in clathrin-mediated endocytosis [18], [19]. Additionally, the Tetherin cytoplasmic tail has a serine-threonine-serine stretch and lysine residues that were identified as targets for HIV-1 Vpu-mediated tetherin downregulation through the recruitment of cellular ubiquitin ligase complexes [20]–[22].

A number of studies have examined the ability of Tetherin to function as a cellular restriction factor; however, there is evidence that Tetherin has additional roles that may be important both in the presence and absence of viral infection. Tetherin was originally described as a marker on differentiated B cells and was suggested to be involved in B cell development [23], [24]. It is apparent now that Tetherin is constitutively expressed on various other cells of the immune system including macrophages, plasmacytoid dendritic cells (pDCs) and T lymphocytes [25], [26]. Tetherin is also preferentially expressed on the surface of various transformed and metastatic cell types [27], [28], though whether Tetherin is actively contributing to the transformed state of the cell is unknown. Additionally, Tetherin has been proposed to act as a scaffold protein in polarized cells [29] and contribute to monocyte adhesion to endothelial cells [30]. Apart from directly restricting viral egress, Tetherin has been shown to have immunomodulatory properties. Tetherin regulates cytokine secretion in murine [25] and human pDCs. In human pDCs, Tetherin acts as a ligand for immunoglobulin-like transcript 7, which results in modulation of Toll-like receptor-mediated IFN and proinflammatory cytokine secretion [31]. Additionally, a cDNA screen identified BST-2 (aka Tetherin) as one of several uncharacterized cellular factors that could activate NF-κB [32]. However, this potential role for Tetherin in signaling has not been further explored.

Here we identify a previously undescribed short isoform of human Tetherin generated by alternative translation initiation at an in-frame codon 33 nucleotides downstream of the canonical translation start site which results in the production of two Tetherin species. The long (l-) and short (s-) Tetherin isoforms are generated in both cultured and primary human cells. Importantly, the two isoforms have distinct biological properties. s-Tetherin, which lacks 12 residues present in the long isoform, is significantly more resistant to HIV-1 Vpu-mediated downregulation and consequently more effectively restricts HIV-1 release in the presence of Vpu than l-Tetherin. In contrast, the ebolavirus glycoprotein (GP) effectively counters both Tetherin isoforms. We also find that Tetherin is a significant activator of NF-κB. However, the Tetherin isoforms have dramatically different signaling potential, with NF-κB activation requiring a tyrosine motif found in the l-Tetherin isoform but absent in s-Tetherin. These observations provide insight into how the expression of alternatively translated Tetherin isoforms regulates multiple cellular functions and begins to shed more light on a poorly characterized signaling property.

## Results

### Alternative translation initiation produces two isoforms of Tetherin

**Figure 1:**
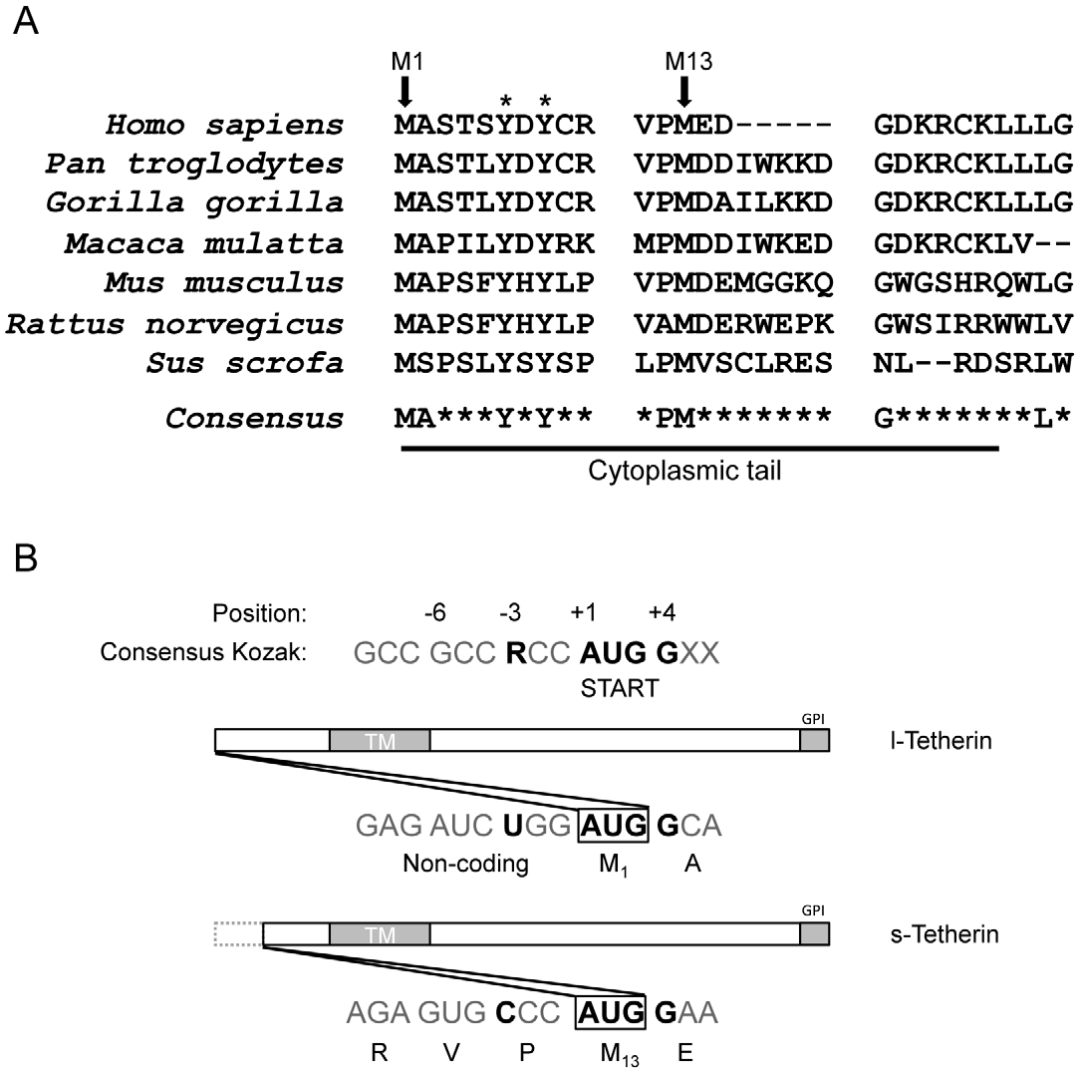
Alignment of Tetherin sequences and comparison to a consensus Kozak translation initiation sequence. (A) Amino acid alignment comparing the amino-terminal cytoplasmic region of Tetherin from various mammalian species. Accession numbers- *Homo sapiens*: NP_004326, *Pan troglodytes*: NP_001177409 XP_552491, *Gorilla gorilla*: ADI58594, *Macaca mulatta*: ACV96781, *Mus musculus*: NP_932763 XP_134266, *Rattus norvegicus*: NP_937767 XP_579725, *Sus scrofa*: NP_001155227 (CL Sequencer; 80% limit). Highlighted conserved residues include two cytoplasmic methionine residues, M1 and M13 (arrows), and a previously characterized dual tyrosine motif (*). (B). Comparison of the nucleotide sequences at the M1 and M13 AUGs in human tetherin mRNA to a consensus Kozak translation initiation sequence. Important residues at the −3 and +1 to +4 positions are highlighted in bold black text. Human tetherin does not conform to the consensus at the −3 position. l-Tetherin and s-Tetherin refer to proteins initiated at M1 or M13 respectively. R = Purine.

Sequence analysis of the cytoplasmic tail of Tetherin revealed minimal amino acid conservation across species. Residues that were highly conserved included two cytoplasmic methionine residues and a dual tyrosine motif (Figure 1A), previously found to be involved in Tetherin trafficking between the plasma membrane and intracellular compartments [18], [19]. Methionine codons can function as translation start sites when they are flanked by a Kozak consensus sequence, which influences ribosomal binding at the site of translation initiation in eukaryotic cells. Analysis of the human tetherin mRNA sequence revealed that the Kozak sequences flanking the first AUG (M1) appears to be a potentially “leaky” signal due to a non-consensus pyrimidine at the −3 position (Figure 1B) [33]. The downstream methionine (M13) appears to be a stronger translation start site with compensatory cytosine residues at positions −1 and −2, which could enhance the strength in the absence of the purine in the −3 position [34]. Although neither of these methionine residues can be defined as strong Kozak consensus start sites, both sequences display conservation at the important +1 through +4 positions. Analysis of the mRNA sequences of Tetherin from other species revealed a similar potentially “leaky” Kozak signal for the upstream methionine (Figure S1), suggesting that production of multiple isoforms may be a conserved Tetherin characteristic.

**Figure 2:**
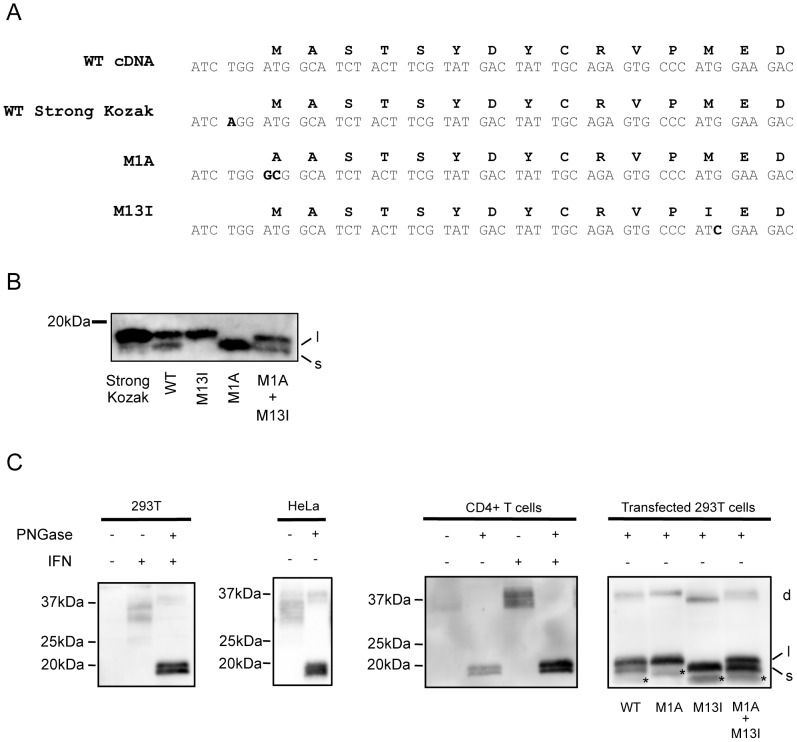
Tetherin exists as two isoforms. (A) Alignment of the Tetherin amino acid (Black text) and cDNA (Grey text) sequences compared to strong Kozak sequence, M1A and M13I point mutations (highlighted in black). (B) HT1080 cells transiently transfected with Tetherin encoding plasmids (WT, Strong Kozak, M1A, M13I or M1A+M13I) were lysed, treated with glycosidase (PNGase) to remove carbohydrate modifications and analyzed by Western blot using a polyclonal Tetherin antibody. l = long isoform, s = short isoform (C) Tetherin isoform expression in cell lysates from IFN stimulated and unstimulated 293T (1^st^ panel), unstimulated HeLa (2^nd^ panel) and primary CD4 T (3^rd^ panel) cells. Deglycosylated tetherin profiles are compared to those from transiently expressing 293T cells (4^th^ panel panel). l- and s- indicate the long and short Tetherin isoforms. Stable tetherin dimers (d) and an uncharacterized smaller molecular mass species (*) often observed under transient transfected conditions are indicated.

To address whether alternatively initiated Tetherin isoforms could be produced, mutations that individually abrogated each of the AUG codons were generated in the wt cDNA (Figure 2A). N-linked glycosylation of Tetherin produces a heterogeneous population of proteins that is not well resolved by electrophoresis [[35] and Figure S2], complicating unambiguous identification of the observed Tetherin bands. Therefore, to better differentiate the potential isoforms that are predicted to differ by 12 residues (∼1.5 kDa), lysates from transiently expressing cells were treated with Peptide: N-Glycosidase F (PNGase) to remove N-linked glycosylation before analysis by Western blot. Analysis of deglycosylated lysates from HT1080 cells expressing the wt Tetherin cDNA reveals two major bands of between 15–20 kDa (Figs. S2 and 2B); the predicted sizes of proteins initiated at the M1 and M13 translation initiation sites is approximately 18 and 16.5 kDa respectively. Transient expression of the wt tetherin cDNA produces more of the larger form compared to the smaller species. Expression of the M13I mutant in HT1080 cells produces a single major product that aligns with the larger protein species seen in wt cDNA transfected cells (Figure 2B). The M1A mutant, that abolishes the upstream potential initiation site, generates a protein that migrates similarly to the smaller species produced by wt cDNA. Co-transfection of plasmids expressing the M1 and M13 mutants recapitulates the pattern seen with wt Tetherin (Figure 2B). Overall, these data demonstrate that transient expression of the wt cDNA produces two alternatively initiated species that we refer to as the l-Tetherin (long) and s-Tetherin (short) isoforms.

To address whether expression of the s-Tetherin isoform was due to translational leakage, an optimized translational initiation sequence was generated by converting the sequence at the −3 position of the upstream M1 ATG in the wt Tetherin cDNA from a pyrimidine (T) to a purine (A). Upon introduction into HT1080 cells of a vector with a strong Kozak sequence at the M1 AUG, expression of l-Tetherin was greatly enhanced while expression of s-Tetherin was almost undetectable (Figure 2B). This mutational analysis, coupled with the data presented above, demonstrates that Tetherin utilizes a non-canonical Kozak sequence to produce alternatively initiated proteins with different cytoplasmic tails.

To determine whether both isoforms of Tetherin are endogenously expressed, non-stimulated and interferon (IFN)-stimulated 293T, HT1080, HeLa, primary human CD4 T-cell lysates were analyzed as described above and compared to 293T cells transiently expressing the M13I and M1A mutants. As predicted from previous results, no Tetherin was seen upon analysis of deglycosylated proteins from unstimulated 293T and HT1080 cells. Interferon treatment of 293T and HT1080 cells induces Tetherin expression and upon PNGase treatment, collapses a diffuse banding pattern to two bands that migrate similarly to the two isoforms produced by transfection of l-Tetherin and s-Tetherin encoding plasmids (Figure 2C, 1^st^ panel and Figure S3). This demonstrates that IFN stimulation produces both alternatively initiated forms of Tetherin. Analysis of lysates from HeLa cells, which constitutively express relatively high levels of Tetherin, revealed the presence of the doublet in the absence of IFN (Figure 2C, 2^nd^ panel). Both bands appear to be enhanced after IFN stimulation (Figure S3). Similarly to HeLa cells, analysis of untreated primary CD4 T-cells revealed two Tetherin species that were enhanced upon IFN stimulation (Figure 2C, 3^rd^ panel). In all cell types tested for endogenous expression of Tetherin, roughly equal expression of both isoforms was observed after 48 h of IFN stimulation. Expression of the M13I and M1A mutants in 293T cells produced the l- and s-isoforms seen above in HT1080 cells, however in this case smaller molecular mass species are seen (denoted by an asterisk on the 4^th^ panel of Figure 2C and throughout the manuscript). These species are only observed in transiently expressing 293T, and not in transfected HT1080 or interferon induced cells. Though the derivation of these species is unclear, they may represent a precursor or proteolyzed product. Finally, analysis of cells transiently expressing rhesus or murine Tetherin cDNA or IFN treated murine J774 cells demonstrated a doublet indicative of production of two isoforms (Figure S4A and B) indicating that production of long and short isoforms of Tetherin is a highly conserved feature. In contrast, the rhesus cell line FRhK-4 appears to produce only a single Tetherin isoform (Figure S4B). Taken as a whole, the presence of these isoforms in numerous cell types, under various conditions and in multiple species is suggestive of important biological roles for the newly identified l-and s-Tetherin isoforms.

### Tetherin isoforms assemble into homo- and heterodimers

Tetherin exists as a disulfide-linked dimer that is believed to represent the functional form necessary for viral restriction. Structural studies on the ectodomain suggest that Tetherin dimers may also form higher order aggregates, which may be functionally important [36], [37]. To address whether the isoforms interact to form heterodimers, lysates from HT1080 cells expressing wt, l- or s-Tetherin were separated under non-reducing conditions and analyzed by Western blot. Expression of either the l- or s-Tetherin isoform produced single bands of a molecular mass indicative of homodimer formation (Figure S5A). In comparison, the wt cDNA produced a broader expression profile that overlapped with the l- and s-profiles suggesting both homo- and heterodimer formation (Figure S5A). To directly assess the interaction of the l- and s- isoforms, a C-terminally FLAG-tagged l-Tetherin and N-terminally AU1 tagged s-Tetherin were co-expressed and immunoprecipitated using antibodies to the epitope tags (Text S1). As anticipated for efficient heterodimer formation, immunoprecipitation with either tag effectively co-precipitated the other isoform (Figure S5B). As discussed below, the ability to form homo- and heterodimers may have significant effects upon the biological activities of Tetherin.

### Both Tetherin isoforms restrict particle release, but differ in their sensitivities to viral antagonists

**Figure 3:**
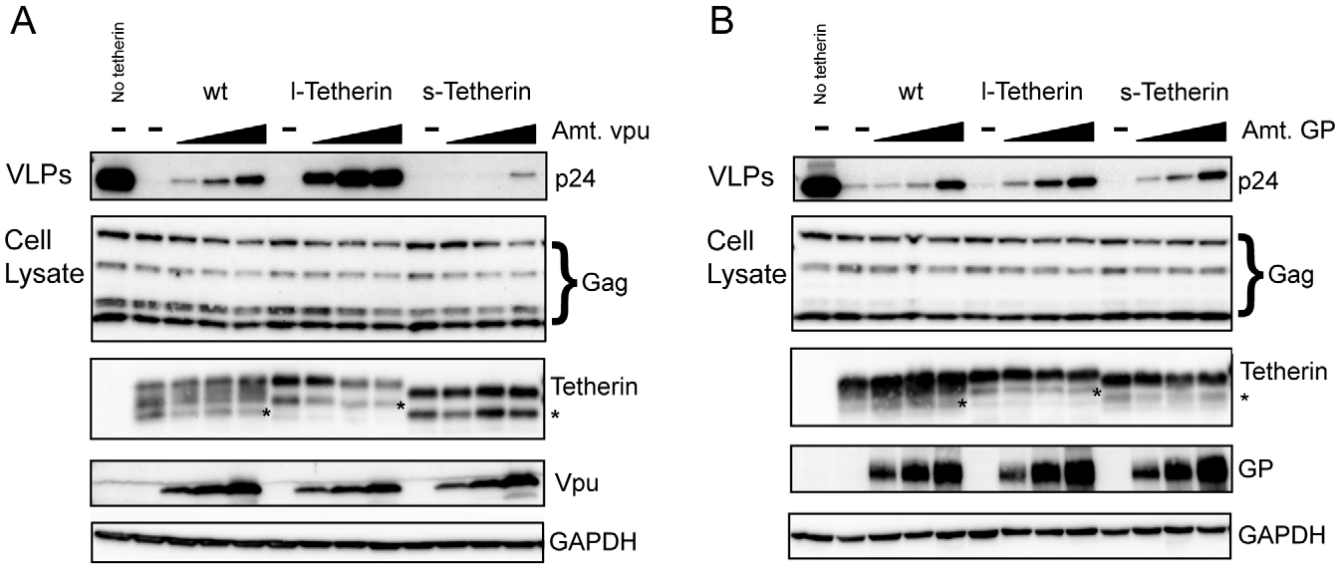
Tetherin isoforms have different sensitivities to viral antagonists. (A and B) 293T cells were transfected with a constant amount of an HIV-1 Gag-Pol expression vector, a constant amount of the indicated Tetherin expression plasmid and increasing amounts of plasmids expressing the viral antagonists (A) Vpu or (B) ebolavirus GP. VLPs (Top panels) from supernatants were purified and analyzed for HIV-1 Gag p24 release. Cell lysates were probed for cellular levels of HIV Gag and Tetherin. As in Figure 2C (*) indicates an undefined species often seen upon transient Tetherin expression in 293T cells. Titration of viral antagonists was assessed using Vpu and GP specific antibodies. GAPDH is used as a lysate loading control.

The cytoplasmic tail of Tetherin is dispensable for function as a viral restriction factor [17]. However, residues in the cytoplasmic tail have been shown to confer sensitivity to viral antagonists. For example, a serine-threonine-serine sequence which is unique to l-Tetherin has been found to be important for efficient Vpu antagonism [22]. Using a transient HIV-1 virus-like particle (VLP) budding assay, the l- and s- isoforms were analyzed for their ability to restrict viral release. Both isoforms were effective viral restriction factors and displayed strong inhibition of HIV-1 VLP release (Figure 3A, top panel). The antiviral activity of wt Tetherin, which expresses both Tetherin isoforms, is antagonized by co-expression of HIV-1 Vpu with VLP release correlating with the level of Vpu expression. In contrast, antiviral activity of the l-Tetherin isoform is exquisitely sensitive to Vpu with significant rescue of viral budding seen at the lowest levels of Vpu expression analyzed (Figure 3A, top panel). Additionally, a decrease in l-Tetherin protein levels was readily observed at higher levels of Vpu expression (Figure 3A, third panel). Conversely, the s- isoform appears to be resistant to antagonism by Vpu, with very little VLP budding rescue observed at the highest levels of Vpu tested (Figure 3A, top panel). Moreover, turnover of the s- isoform also appears to be resistant to Vpu as there was no obvious decrease in s-Tetherin expression even at the highest levels of Vpu (Figure 3A, third panel).

To determine whether the differential sensitivity to viral antagonists was a common property of the Tetherin isoforms, sensitivity to a different viral factor, the ebolavirus glycoprotein, GP, was analyzed. GP is hypothesized to rescue virus budding in Tetherin-expressing cells through a non-cytoplasmic tail-associated mechanism [38] and might therefore be predicted to interact similarly with both Tetherin isoforms. In contrast to the results with Vpu, within the range of GP levels tested, GP-mediated rescue of budding was similar for wt and the two Tetherin isoforms. (Figure 3B, top panel). Additionally, total cellular Tetherin protein levels appeared unchanged for wt Tetherin and each individual isoform even at the highest levels of ebolavirus GP co-expression (Figure 3B, third panel).

**Figure 4:**
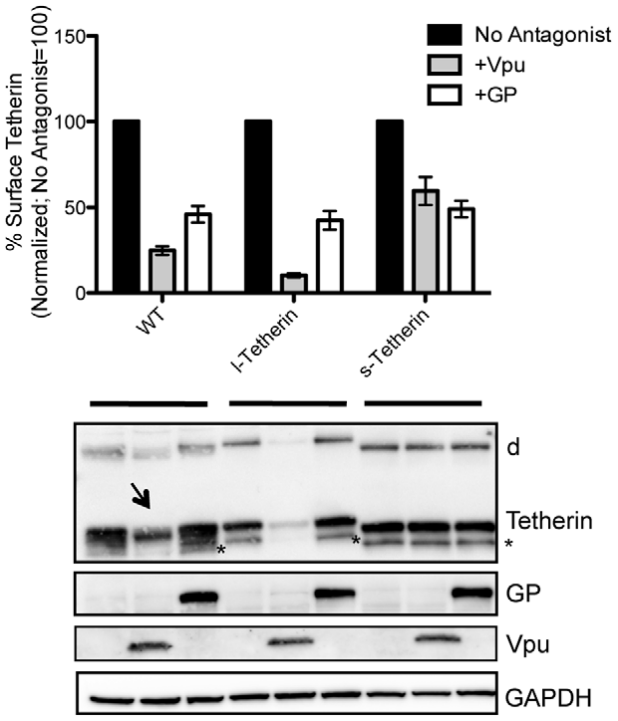
Effect of viral antagonists on surface levels of Tetherin. 293T cells were transiently co-transfected with the indicated Tetherin plasmids in the presence viral antagonists Vpu or ebolavirus GP. Half the cells were analyzed by flow cytometry. Graph represents of the mean MFI from multiple experiments (n = 5); error bars = SEM. Surface Tetherin in the absence of viral antagonist was set to 100%. Expression of total cellular Tetherin was analyzed by Western blot of PNGase treated lysates from cells not used for flow cytometry. Reduced monomers, stable dimmers (d) and an unknown species (*) are indicated. Arrow indicates s-Tetherin isoform seen in wt+Vpu expressing cells. GAPDH used as a loading control.

To determine whether the differential particle release by the isoforms in response to Vpu was due to altered Tetherin cell surface levels, flow cytometric analysis of live cells stained for Tetherin was performed. In Vpu expressing cells, surface wt Tetherin expression followed the same trend as the total cell Tetherin protein levels (compare Figure 3A third panel and Figure 4). Surface expression of l-Tetherin was downregulated by Vpu to a greater extent than the s-Tetherin isoform, while wt Tetherin appeared to have an intermediate level of downregulation (Figure 4). Analysis of lysates from the cells prepared in parallel with those used for flow cytometry confirmed the enhanced degradation of l-Tetherin compared to s-Tetherin observed above in both the reduced monomers and stable dimers. Additionally, preferential degradation of the l-isoform was seen in lysates of cells expressing wt Tetherin and high levels of Vpu (Figure 4; arrow). In comparison, there is no difference in the effect of ebolavirus GP on surface or total levels for the l- and s- isoforms though a decrease in surface staining was observed across all Tetherin constructs tested. The minimal 2-fold decrease in the mean fluorescence intensity of Tetherin surface staining observed in ebolavirus GP expressing cells is consistent with steric shielding of epitopes previously documented for this glycoprotein [39], [40] and appears similar to the slight decrease noted recently by Lopez and colleagues [38]. Overall, these observations suggest that the Tetherin isoforms can have different sensitivities to viral antagonists, which may have important consequences during natural infection.

### Resistance to Vpu antagonism requires mutations in the tyrosine and serine/threonine motifs in Tetherin

**Figure 5:**
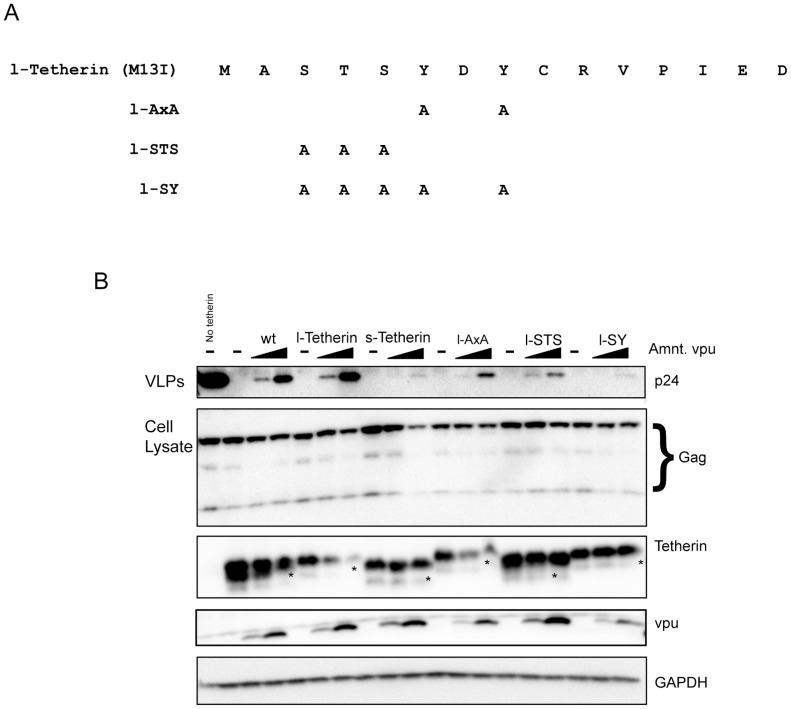
Resistance to Vpu antagonism requires mutations in the tyrosine and serine/threonine motifs in Tetherin. (A) Amino acid sequences of l-Tetherin mutants that disrupt the tyrosine motif (l-AxA), the serine/threonine residues (l-STS) and the combined mutant (l-SY). (B) 293T cells were transfected with HIV-1 Gag-Pol and Tetherin expression vectors plus increasing amounts of plasmids expressing the viral antagonist Vpu (25 or 100 ng). VLPs (Top panels) from supernatants were purified 48 h post transfection and analyzed for HIV-1 Gag p24 release by Western blot. Cell lysates were probed for cellular levels of HIV-1 Gag and Tetherin. As in Figure 2C (*) indicates an unknown species often seen upon transient Tetherin expression in 293T cells. Titration of Vpu was detected using anti-Vpu antibody. GAPDH is used as a lysate loading control.

To further explore the basis for the relative Vpu resistance of s-Tetherin compared to l-Tetherin, a series of mutations were engineered into the 12 residue region unique to l-Tetherin (Figure 5A). Two motifs within this region have been proposed to play a role in Vpu mediated antagonism; a dual tyrosine motif (amino acids 6 and 8, Figure 5A) involved in endocytic cycling of Tetherin [41] and a stretch of serine and threonine residues (amino acids 3 to 5, Figure 5A) that can be ubiquitinated [22], [42]. Analysis of HIV-1 VLP release from 293T cells transiently expressing a tyrosine mutant AxA produced in l-tetherin expressing cDNA shows decreased release in response to Vpu as compared to parental l-Tetherin (lane l-AxA, Figure 5B). However, the observed decreased Vpu antagonism of AxA is not as pronounced as that seen with s-Tetherin. Altering the serine/threonine residues also impairs Vpu mediated particle release (lane l-STS, top panel Figure 5B). However, as with the tyrosine mutations the effect seen is intermediate between l- and s-Tetherin. A mutant that combines the tyrosine and serine/threonine changes appears to recapitulate the high level of resistance to Vpu antagonism observed with s-Tetherin (lane l-SY, top panel Figure 5B). Thus it appears that both these regions are involved in Vpu mediated antagonism of Tetherin function.

Although the tyrosine and serine/threonine mutants appear to similarly affect response to Vpu antagonism and particle release, the STS mutant Tetherin protein demonstrates resistance to Vpu-mediated degradation comparable to that of s-Tetherin (third panel, Figure 5). In contrast, the AxA mutant protein levels decrease upon Vpu expression comparably to parental l-Tetherin (third panel, Figure 5). The combined tyrosine and serine/threonine mutant (third panel, lane l-SY) is Vpu resistant like s-Tetherin and the STS mutant. Overall, these findings support an important role for the STS region in Vpu mediated degradation of Tetherin.

### The l-Tetherin isoform is an inducer of NF-κB

**Figure 6:**
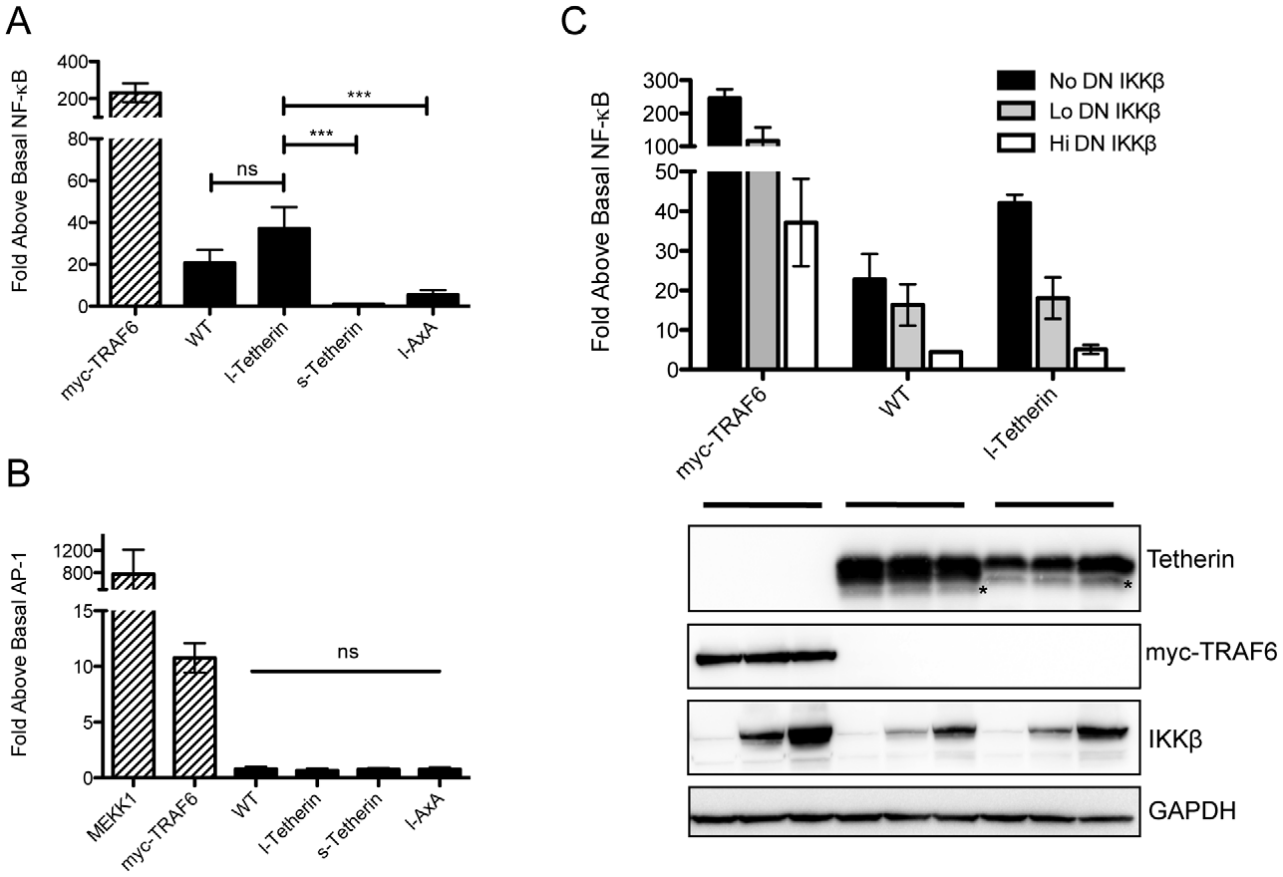
Tetherin isoforms differentially activate NF-κB. (A) 293T cells transiently co-transfected with either a wt, l-Tetherin, s-Tetherin or l-AxA encoding plasmid plus an NF-κB responsive firefly luciferase reporter plasmid were lysed and analyzed for luciferase activity 24 h post transfection. Myc-TRAF6 used as a positive control for NF-κB activation. NF-κB signaling experiments (n = 8 in triplicates) were analyzed by one-way ANOVA. (B) Luciferase assay for AP-1 activation was assessed as in (A) using an AP1 responsive firefly luciferase plasmid (500 ng) in the presence of the same Tetherin encoding plasmids. MEKK1 and myc-TRAF6 were used as positive controls for AP1 activation. AP1 signaling experiments (n = 4 in triplicates) were analyzed by one-way ANOVA. (C) Wt and l-Tetherin were co-transfected with increasing amounts of a FLAG epitope tagged dominant negative (DN)-IKKβ and the NF-κB responsive luciferase reporter. Myc-TRAF6 was used as a positive control (n = 3 in triplicates). Representative blot showing expression of co-transfected constructs as well as a GAPDH loading control. On the Western blots, as in Figure 2C, (*) indicates unknown species. In the graphs ns = not significant; *** p<0.001.

Tetherin has been primarily characterized as a cell intrinsic viral restriction factor. However, Tetherin has been proposed to have additional activities including a role in induction of the proinflammatory response regulator NF-κB [32]. To confirm that Tetherin was able to activate NF-κB, a luciferase reporter assay was employed in 293T cells transiently expressing Tetherin. Activation of NF-κB by Tetherin displays a bell shaped curve with decreased stimulation at high levels of Tetherin expression (Figure S6), suggesting an optimal range of expression for signaling. Expression of wt Tetherin led to NF-κB activation approximately 20–30 fold over control cells transfected with the expression vector alone (Figure 6A). Moreover, Tetherin activation of NF-κB is specific since there was no effect upon AP1-regulated signaling (Figure 6B). TRAF6, a ubiquitin ligase involved in regulating various signal transduction pathways including NF-κB, upregulated both NF-κB and AP1. Supporting the specificity of the observed effect for NF-κB, activation by Tetherin was effectively blocked in a dose dependent manner by expression of a dominant-negative form of the NF-κB activating kinase IKKβ (DN-IKKβ, Figure 6C).

We next assessed whether the Tetherin isoforms had differential abilities to activate NF-κB. Unlike wt Tetherin, the truncated s-Tetherin isoform displayed no NF-κB activation (Figure 6A). Conversely, compared to wild type, the l-Tetherin isoform was, on average, a more potent activator and increased NF-κB activity approximately 40-fold over basal levels. As was seen for wt Tetherin, NF-κB activation by l-Tetherin was sensitive to inhibition by DN-IKKβ (Figure 6C). These findings demonstrate that sequences within the 12 amino acid region absent in s-Tetherin are required for the signaling events downstream of Tetherin that activate NF-κB.

### NF-κB activation requires the tyrosine motif and is negatively regulated by s-Tetherin

**Figure 7:**
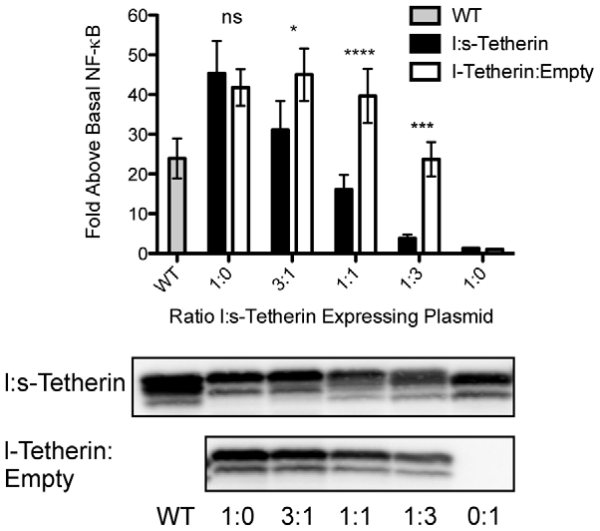
s-Tetherin modulates l-Tetherin-mediated NF-κB induction. 293T cells were transiently transfected with an NF-κB luciferase reporter and varying ratios of l- and s-Tetherin expression vectors with the total tetherin expression plasmids amount kept constant. Cells were lysed and analyzed for luciferase activity. In parallel, an l:s titration (black bars) was compared to l-Tetherin titrated with empty plasmid (white bars). wt Tetherin signaling (grey bar) was assessed during each experimental replicate (n = 9 in triplicate). Two-way ANOVA performed to assess statistical significance. A representative Western blot for Tetherin is shown for each set of titrations. *p<0.05; ***p<0.001, **** p<0.0001.

As described above, Tetherin can assemble into hetero and homodimers of the l- and s- isoforms. Given the observations that s-Tetherin does not activate NF-κB, l-Tetherin is more potent than wt, and wt Tetherin has an intermediate signaling phenotype to that of l- and s-isoforms, we investigated whether the short isoform could modulate signaling activity of the longer species. To address this question, varying ratios of the two isoforms were assessed for their ability to activate NF-κB. As seen in Figure 7, l-Tetherin mediated NF-κB activation is strongly diminished by the presence of s-Tetherin. The ability of s-Tetherin to sharply reduce signaling at a 1∶1 and 1∶3 ratio of l- to s-Tetherin expressing plasmids is supportive of a model in which homo-oligomers of the longer species are needed to signal. Overall, this observation suggests an inhibitory role for s-Tetherin in regulating NF-κB signaling by l-Tetherin.

Within the 12 amino acids unique to l-Tetherin are two highly conserved tyrosine residues at positions 6 and 8 (Figure 1). To address whether these residues contributed the ability of the longer isoform to activate NF-κB, alanine substitution mutations were generated in the context of l-Tetherin and tested by transient expression in 293T cells. Compared to the parent l-Tetherin, the ability of the tyrosine mutant (AxA) to activate NF-κB was significantly reduced (Figure 6A). Although impaired, a low but reproducible level of activity was observed for the dual tyrosine mutant. These data suggest that the mutated residues form part of an important signaling motif within the cytoplasmic tail region needed to activate NF-κB that is present only in the longer isoform of Tetherin.

## Discussion

Here we identify a previously undescribed isoform of human Tetherin generated by translation at an in-frame initiation codon 33 nucleotides downstream of the canonical translation start site, which results in the loss of a 12-amino-acid N-terminal sequence. Alternative translation initiation in eukaryotes can occur by 3 different mechanisms: internal ribosome entry, reinitiation, or leaky ribosome scanning. Our analysis indicates that the tetherin mRNA contains a “leaky” Kozak sequence around the first AUG codon (M1). Based on the scanning model of translation, this would allow the ribosome to skip over the first start codon a fraction of the time, leading to initiation at the next in frame AUG (M13). Selective disruption of each AUG and introduction of a strong Kozak at the upstream AUG confirmed that alternate sites of translation initiation accounted for each of the observed products. Conservation of an upstream leaky Kozak and a second cytoplasmic methionine in most mammalian tetherin sequences, along with our analysis of expression from rhesus and murine cDNAs, suggests that multiple isoforms exist in other species and indicate an important biologic role(s) for the two isoforms.

For many messages with alternatively translated isoforms the resultant proteins display distinct functionalities [43]–[46]. This is clearly the case for Tetherin where we identified unique biologic properties for the two isoforms. Although both isoforms exhibit antiviral activity, the shorter Tetherin isoform, derived by initiation at M13, is highly resistant to the antagonistic effects of HIV-1 Vpu and therefore may play a more important role in restricting virion budding in HIV-1 infected cells. Analysis of mutants in the 12 residues unique to l-Tetherin suggests that the combined loss of two tyrosine and three serine/threonine residues accounts for the Vpu resistance of s-Tetherin. By contrast, l-Tetherin seems to be hypersensitive to Vpu antagonism. However, this longer isoform possesses the ability to induce the immune response regulatory transcription factor NF-κB while s-Tetherin does not have this capability. Expression of the wt Tetherin cDNA, which produces both protein species yielded phenotypes for Vpu sensitivity and NF-κB signaling that were intermediate between those seen upon expression of the individual isoforms. This likely reflects that fact that when the isoforms are co-expressed both hetero and homodimers form, and that the unique properties observed (e.g. Vpu resistance and NF-κB signaling) require short-short or long-long homodimers respectively.

In several other systems where alternative translation initiation produces multiple isoforms there are observed differences in the ratios of the isoforms in tissues [44], [45] or under various physiologic conditions [47] suggesting regulation of alternative initiation. For example, the levels of glucocorticoid receptor (GR) isoforms vary significantly in different tissues, indicating that cells can regulate this ratio with important consequences for GR gene regulation [44]. Similarly, LPS regulates differential expression of alternatively translated forms of the CCAAT/enhancer-binding *trans*-activator proteins C/EBPα and C/EBPβ, producing isoforms with unique properties [47]. In our experiments, the ratios of l- and s-Tetherin differed in transient expression versus endogenous expression. Lysates harvested 48 h post-transient transfection appeared to produce more of the longer species. In contrast, 48 h stimulation with IFN produced roughly equal amounts of both isoforms. Interestingly, tissue specific expression of ovine Tetherin isoforms has been reported [6]; however, the mechanism for generation of these isoforms was not investigated. Our analysis of human Tetherin did not query a large number of cell types or conditions, therefore it will be interesting to more fully address whether there are instances in which the ratio of l- and s-Tetherin is regulated. Conversely, the increased sensitivity of l-Tetherin to Vpu compared to s-Tetherin suggests that viral antagonists might alter the isoform ratio. Because both NF-κB signaling and Vpu sensitivity appear to be affected by the formation of homodimers, this suggests a model whereby subtle changes in isoform stoichiometry, either by differential expression or susceptibility to viral antagonists of one isoform, could rapidly and dramatically affect Tetherin function.

The importance of the shorter Tetherin isoform with increased antiviral activity or resistance to viral antagonists is strongly supported by recent evidence. While this manuscript was in preparation, a polymorphic allele that abrogated expression from the first methionine residue, and thus produced only short isoforms, was described in NZW mice. In these mice, expression of shorter Tetherin isoforms strongly correlated with decreased Friend retrovirus replication and pathogenesis [48]. Tetherin sequence variants have also been identified in rhesus macaques, African green monkeys and mice [49], [50]. Interestingly, none of the identified macaque polymorphisms account for the single species we observed by Western blot in the macaque FRhK-4 cell line. Further analysis of genomic sequence of tetherin from this rhesus cell line is required to determine the form being expressed.

We find that the ability of s-Tetherin to retain budding HIV-1 virions and to resist Vpu more effectively than l-Tetherin appears to be due to loss of tyrosine and serine threonine motifs found in the cytoplasmic tail of the long isoform. Mutation in either motif rendered Tetherin partially resistant to Vpu. Combining these two sets of mutations recapitulated loss of Vpu sensitivity seen for s-Tetherin suggesting these represent important differences between l- and s-Tetherin. The tyrosine motif in l-Tetherin is a non-canonical trafficking signal (YXYXXV) that engages the AP-1 and AP-2 clathrin adaptors [18], [19]; mutations within this sequence alter cellular trafficking and distribution [51], [52]. It has recently been demonstrated that Vpu impairs recycling of Tetherin to the cell surface and that this is an key mechanism for antagonizing Tetherin function [41]. It will be interesting to determine if s-Tetherin effectively recycles in the presence of Vpu or if endocytosis from the cell surface is affected. Also, within the region unique to l-Tetherin is a serine-threonine-serine stretch that has been shown to be important for Vpu mediated Tetherin degradation [22] although more recent studies have questioned this conclusion [22], [42]. Our finding that mutations in this motif confer partial resistance to Vpu antagonism and also affect Vpu induced degradation support an import role for this region in interactions with Vpu. Interestingly, particle retention and Tetherin degradation appear to be separable features because the STS mutant appeared to be resistant to Vpu induced degradation while the YxY mutant remained sensitive to Vpu degradation, yet both mutations partially impair Vpu induced virion release. Our data is consistent with a model in which both decreased protein turnover and altered trafficking account for the ability of s-Tetherin to effectively retain virions in Vpu-expressing cells.

The observation that the long isoform activates NF-κB while the short isoform shows no activity suggests that sequences within the 12 residues unique to l-Tetherin mediate signaling. Moreover, data demonstrating higher NF-κB induction by expression of the l-Tetherin isoform compared to the wt cDNA, coupled with an inhibitory affect of s-Tetherin on NF-κB activation, support a model in which homodimers of the longer isoform are responsible for signaling. Though Tetherin does not possess canonical tyrosine-based motifs involved in activating signal transduction pathways, there is evidence that dimerization of CLEC-2 allows approximation of a non-canonical tyrosine-based motif and permits signaling via Syk kinase [53]. Furthermore, phosphoproteomic analysis has shown that Tetherin tyrosine residues at positions 6 and 8 can be phosphorylated [54]. Moreover, mutation of both these tyrosine residues greatly diminished NF-κB induction, demonstrating that they are critical for this activity. Because the dual tyrosine residues have been shown to be important in trafficking it will be crucial to assess whether differential localization may also play a role in activating signal transduction. The ability of a dominant negative form of the kinase IKKβ to inhibit Tetherin-induced NF-κB activity indicates that activation likely involves the canonical pathway, however the mechanism by which Tetherin couples to this pathway remains to be elucidated. Similarly to our findings with Tetherin, it has recently been found that the cytoplasmic tail domain of HIV-1 envelope activates NF-κB, but not AP1 [55]. In this case activation occurs via the canonical NF-κB pathway, with HIV-1 envelope directly engaging TGF-β-activated kinase 1 (TAK1). Whether Tetherin utilizes a similar mechanism to specifically activate NF-κB remains to be explored.

It has been demonstrated that Tetherin is a ligand for ILT7 on human dendritic cells and that binding initiates signaling via the ILT7–FcεRIγ complex, inhibiting production of interferon and proinflammatory cytokines by dendritic cells [31]. It will be interesting to investigate if ILT7, or an unknown ligand can modulate NF-κB activation by Tetherin. The viral restriction factor TRIM5α has recently been shown to function as a unique pattern recognition receptor recognizing the hexagonal lattice pattern of entering retroviral capsids to activate NF-κB and MAP kinase pathways [56]. In a similar vein, we speculate that Tetherin could recognize the “pattern” caused by budding virions to activate NF-κB.

## Materials and Methods

### Cell culture

293T, HT1080 and HeLa cells were maintained in high glucose DMEM+10% Cosmic Calf Serum (Hyclone). Primary CD4 T cells (obtained from the University of Pennsylvania Center for AIDS Research Immunology Core, ND307) were maintained in RPMI+10% FBS (Invitrogen). All cells were maintained at 37°C with 5% CO_2_.

### Plasmids and reagents

Human tetherin cDNA in pCMV-SPORT6 was acquired from Open Biosystems. Tetherin mutants were generated using site-directed mutagenesis by PCR. Primers for site-directed mutagenesis were designed using QuikChange Primer Design Program (Agilent Technologies). The HIV-1 gag-pol encoding construct, psPAX2 was obtained from Addgene (plasmid 12260). HIV-1 vpu and ebolavirus GP cloned into pCAGGS were previously described [3], [57]. The NF-κB reporter plasmid, pBIIX-Luciferase was provided by Dr. Michael May (University of Pennsylvania). The TRAF6 coding region was cloned from a cDNA (Open Biosystems) into pKMyc (Addgene plasmid 19400) to generate an N-terminally FLAG-tagged version using an XbaI restriction site adjacent to the FLAG-tag. The dominant negative FLAG epitope tagged IKKβ (K44M) plasmid was obtained from Addgene (plasmid 11104). The AP1 reporter (Biomyx) and MEKK1-delta plasmids were obtained from Dr. Sunny Shin (University of Pennsylvania).

### Tetherin protein expression analysis

HT1080 cells in a 6 well were transiently transfected with Tetherin expression constructs using Lipofectamine2000 (Invitrogen) according to manufacturers instructions. For IFN induction experiments, HT1080, 293T, HeLa and primary CD4 T cells were incubated +/− 1000 U of recombinant type I IFN (PBL Interferon Source) for 48 h. Two days post-transfection/IFN treatment, cells were washed with PBS and lysed using RIPA buffer (10 mM Tris-HCl pH 8.0, 5 mM EDTA, 140 mM NaCl, 1% sodium deoxycholate, 0.1% SDS, 1% NP40). Lysates were passaged through a 25G needle before being cleared by centrifugation at 17,900× g for 15 min at 4°C. Cleared lysates were treated with either treated or not with PNGase (New England BioLabs) for 2 h, then reduced using DTT containing loading buffer. Samples were separated on a 15% Criterion gel (BioRad) and transferred to PVDF membrane. After blocking in 5% milk TBST (Tris-buffered saline+0.1% Tween), Western blots were analyzed for Tetherin expression using rabbit anti-BST2 sera (Dr. Klaus Strebel, #11721 National Institutes of Health AIDS Research and Reference Reagent Program).

### VLP budding assay

2.5×10^5^ 293T cells seeded on a 24 well plate were co-transfected with the indicated tetherin construct (12.5 ng), an HIV-1 Gag-Pol expression vector pSPAX (50 ng) and increasing amounts of (25, 50 or 100 ng) of pCAGSS-vpu or pCAGGS ebolavirus GP using Lipofectamine2000. Cell lysates and supernatants were harvested 48 h post-transfection. Cell lysates were harvested in Triton X-100 buffer (50 mM Trs-HCl pH 8.0, 5 mM EDTA, 150 mM NaCl, 1% Triton X-100) with Complete (Roche) protease inhibitor cocktail. Cell lysates were cleared by centrifugation at 17,900× g for 3 min at 4°C. Supernatants containing VLPs were cleared are 1700× g for 2 min at 4°C. VLPs were subsequently purified by pelleting through a 20% sucrose cushion at 40,000 in a TLA120.1 rotor (Beckman) for 30 min. VLPs were resuspended in PBS on ice for 3 h. Cleared cell lysates and resuspended VLPs were separated on 15% or 4–15% Criterion gels (BioRad) respectively before being transferred to PVDF membrane. Membranes were blocked in 5% milk in TBST for 40 min prior to incubation with primary antibody. HIV-1 Gag p24 was detected in Western blots for both VLPs and cell lysates using a monoclonal anti-p24 antibody (24-3, National Institutes of Health AIDS Research and Reference Reagent Program). The GAPDH loading control was analyzed using GAPDH antibody (Calbiochem, CB1001). HIV-1 Vpu was detected using HIV-1 pNL4-3 Vpu antiserum (969, National Institutes of Health AIDS Research and Reference Reagent Program). Ebolavirus GP was detected using rabbit antiserum against the GP1 portion of the protein [58].

### Flow cytometry analysis of tetherin cell surface expression

293T cells (2.5×10^5^) seeded on a 24 well plate were transiently co-transfected with 25 ng Tetherin constructs (wt, l-Tetherin, s-Tetherin or l-AxA) and 100 ng HIV-1 vpu or ebolavirus GP. Two days post transfection, cells were washed once with 1×PBS while on the plate. Whole cells were removed using cold 1×PBS and the cells were split into equal aliquots used for flow cytometry and western blot analysis. Cells used for Western blot analysis were pelleted by centrifugation then lysed in Triton X-100 buffer. Lysates were cleared and PNGase treated prior to SDS/PAGE Western blot analysis as described above. Cells to be used for flow cytometry were resuspended in cold FACS buffer (1×PBS, 1%BSA+0.05% sodium azide) and pelleted at 2150× g. Cells were resuspended in FACS buffer+PE conjugated anti-BST2 antibody (Biolegend). After a 1 h incubation on ice, cells were washed three times with cold FACS buffer and analyzed on a FACS Calibur (BD Biosciences Immunocytometry Systems). Data analysis performed using FlowJo 9.3.1 (Tree Star).

### Luciferase assay

293T cells (2.5×10^5^) were seeded on a 24 well plate and the following day were transfected with the indicated tetherin constructs (50 ng) and pBIIX-Luciferase reporter (250 ng) using Lipofectamine2000. In experiments where the long and short isoforms were co-expressed the total amount of tetherin expression plasmid was kept constant at 50 ng. At 30 h post-transfection, cell lysates were harvested in Triton X-100 lysis buffer. Lysates were transferred to a black flat bottom 96-well plate. Luciferase Assay System substrate (Promega E1501) was added to the lysates according to manufacturers directions. Samples were analyzed in a Luminoskan Ascent microplate luminometer (Thermo Scientific). Statistical analysis was performed using PRISM (GraphPad).

## Supporting Information

Figure S1 **Comparison of Kozak translation initiation sequences of mammalian Tetherin messages.** Tetherin cDNA sequences from various mammals (Ensembl Gene IDs: CpzBST2 (Chimp)- ENSPRTRT00000019678, GorBST2 (Gorilla)-ENSGGOT00000015329, RhBST2 (Rhesus)- ENSMMUT00000008172, MsBST2 (Mouse)- ENSMUST00000051672; NCBI Ref Sequence: RtBST2 (Rat)- NM_198134.1) restricted to approximately half the cytoplasmic tail and 9 bases upstream of the canonical start codon were aligned and are shown with the deduced amino acid sequences under the corresponding codons. Matches to important residues in the consensus Kozak sequence (−3, and +1 to +4) are denoted in black in each sequence. In all cases, the upstream methionine deviates from the consensus at the −3 position.
(TIF)

Figure S2 **Resolution of heterogeneous Tetherin expression profile by removal of carbohydrate modification with PNGase.** Lysates from HT1080 cells transiently expressing wt Tetherin cDNA were prepared 48 h post transfection. Lysates were analyzed by Western blot using a Tetherin antibody. Left panel (no PNGase), right panel (with PNGase).
(TIF)

Figure S3 **Endogenous expression of Tetherin isoforms.** IFN stimulated or unstimulated HeLa, HT1080 and 293T cells were analyzed at 48 h post exposure. Cellular lysates were used directly or digested with PNGase and analyzed by Western blot. A lighter exposure of PNGase treated profile is shown below the main blot. HT1080 cells transiently expressing wt, l-Tetherin, s-Tetherin, l+s-Tetherin or wt Strong Kozak mutants were harvested, PNGase treated and analyzed adjacent to endogenous Tetherin samples.
(TIF)

Figure S4 **Expression profiles of rhesus and murine Tetherin.** (A) 293T cells were transfected with murine Tetherin cDNA. Concurrently, murine J774 cells were IFN treated for 48 h. Lysates were PNGase treated and analyzed by Western blot using an anti-mouse CD317 antibody (BioLegend, 127101). Shorter exposure of the transfected 293T cells is shown to the right. (B) Lysates from 293T cells transiently expressing rhesus Tetherin cDNA were analyzed adjacent to lysates of rhesus FRhK-4 cells. The right panel is a lighter exposure of the 293T lane from the same blot. While the J774 cells, the murine and rhesus Tetherin cDNAs produce two isoforms, only a single species that corresponds to the upper (presumably l-Tetherin) isoform is seen in FRhK-4 cells.
(TIF)

Figure S5 **Isoforms produce homo- and heterodimers.** (A) HT1080 cells transiently expressing Tetherin mutants form homodimers. HT1080 transfected with either wt, l-, s- or l+s-Tetherin were lysed in RIPA buffer. Lysates were PNGase treated for 2 h without denaturation. Deglycosylated samples were analyzed under non-reducing conditions and probed for Tetherin using anti-BST2 rabbit sera. l:l Long homodimers; s:s short homodimers. (B) Co-immunoprecipitation of epitope tagged isoforms. Cartoon of differentially tagged l- and s-Tetherin expression vectors with tags adjacent to the GPI anchor additions site or at the amino terminus respectively. Epitope tagged Tetherin isoforms were transiently expressed in 293T cells then RIPA lysates were precipitated using the indicated antibodies. Precipitates were analyzed by SDS/PAGE and Western blot. Lane 1, mock transfected; Lane 2, wt-Tetherin FLAG; Lane 3, l-Tetherin FLAG; Lane 4, AU1 s- Tetherin; Lane 5, l-Tetherin FLAG+AU1 s- Tetherin.
(TIF)

Figure S6 **NF-κB induction varies with Tetherin expression level.** 293T cells (2×10^5^) were transiently transfected with a range of amounts of Tetherin expression plasmid (12.5, 25, 50, 100 or 200 ng) and a constant amount NF-κB luciferase reporter. Total DNA transfected was kept constant by including empty vector. The results consistently show a bell shaped response for NF-κB activation by Tetherin. From these results 50 ng was chosen as an optimal amount of plasmid for the NF-κB activation assays. This graph is a representative experiment done in triplicate, bars = SD.
(TIF)

Text S1 **Supplementary Methods.**
(DOCX)
