# Supplementary figures and images for: Identification of Alternatively Translated Tetherin Isoforms with Differing Antiviral and Signaling Activities
Source: PLoS Pathog. 2012 Sep 27;8(9):e1002931. doi: 10.1371/journal.ppat.1002931 (PMC3460627; doi:10.1371/journal.ppat.1002931)

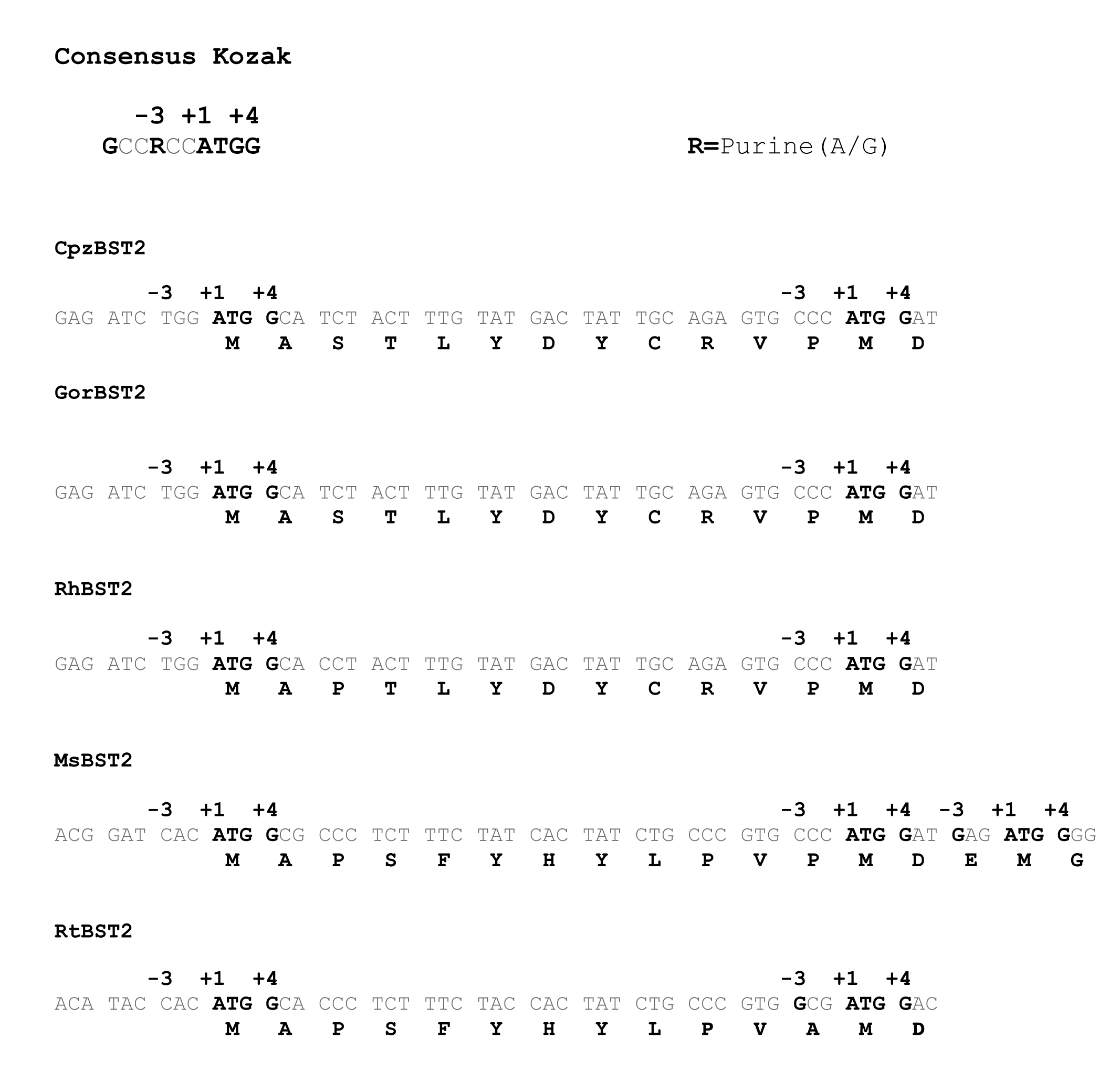

Supplement: Figure S1 — Comparison of Kozak translation initiation sequences of mammalian Tetherin messages. Tetherin cDNA sequences from various mammals (Ensembl Gene IDs: CpzBST2 (Chimp)- ENSPRTRT00000019678, GorBST2 (Gorilla)-ENSGGOT00000015329, RhBST2 (Rhesus)- ENSMMUT00000008172, MsBST2 (Mouse)- ENSMUST00000051672; NCBI Ref Sequence: RtBST2 (Rat)- NM_198134.1) restricted to approximately half the cytoplasmic tail and 9 bases upstream of the canonical start codon were aligned and are shown with the deduced amino acid sequences under the corresponding codons. Matches to important residues in the consensus Kozak sequence (−3, and +1 to +4) are denoted in black in each sequence. In all cases, the upstream methionine deviates from the consensus at the −3 position. (TIF) [file ppat.1002931.s001.tif]

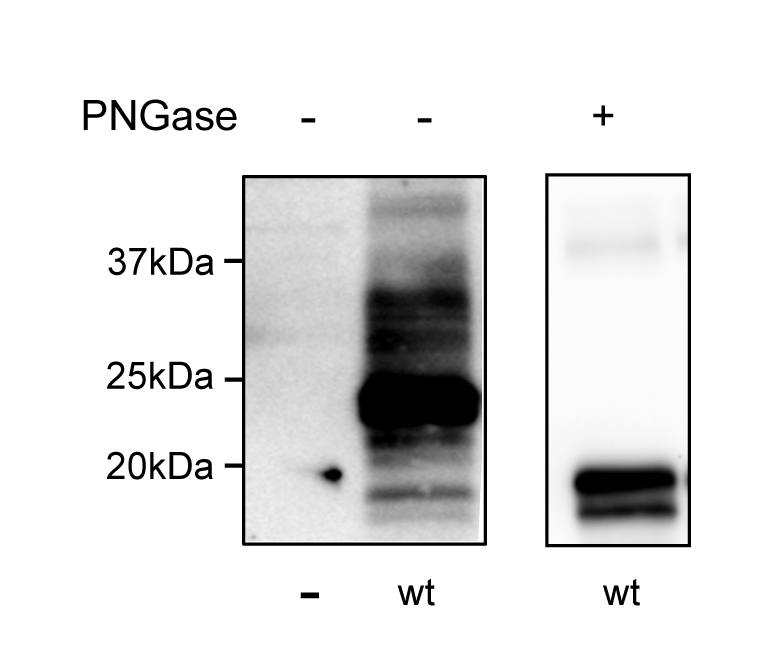

Supplement: Figure S2 — Resolution of heterogeneous Tetherin expression profile by removal of carbohydrate modification with PNGase. Lysates from HT1080 cells transiently expressing wt Tetherin cDNA were prepared 48 h post transfection. Lysates were analyzed by Western blot using a Tetherin antibody. Left panel (no PNGase), right panel (with PNGase). (TIF) [file ppat.1002931.s002.tif]

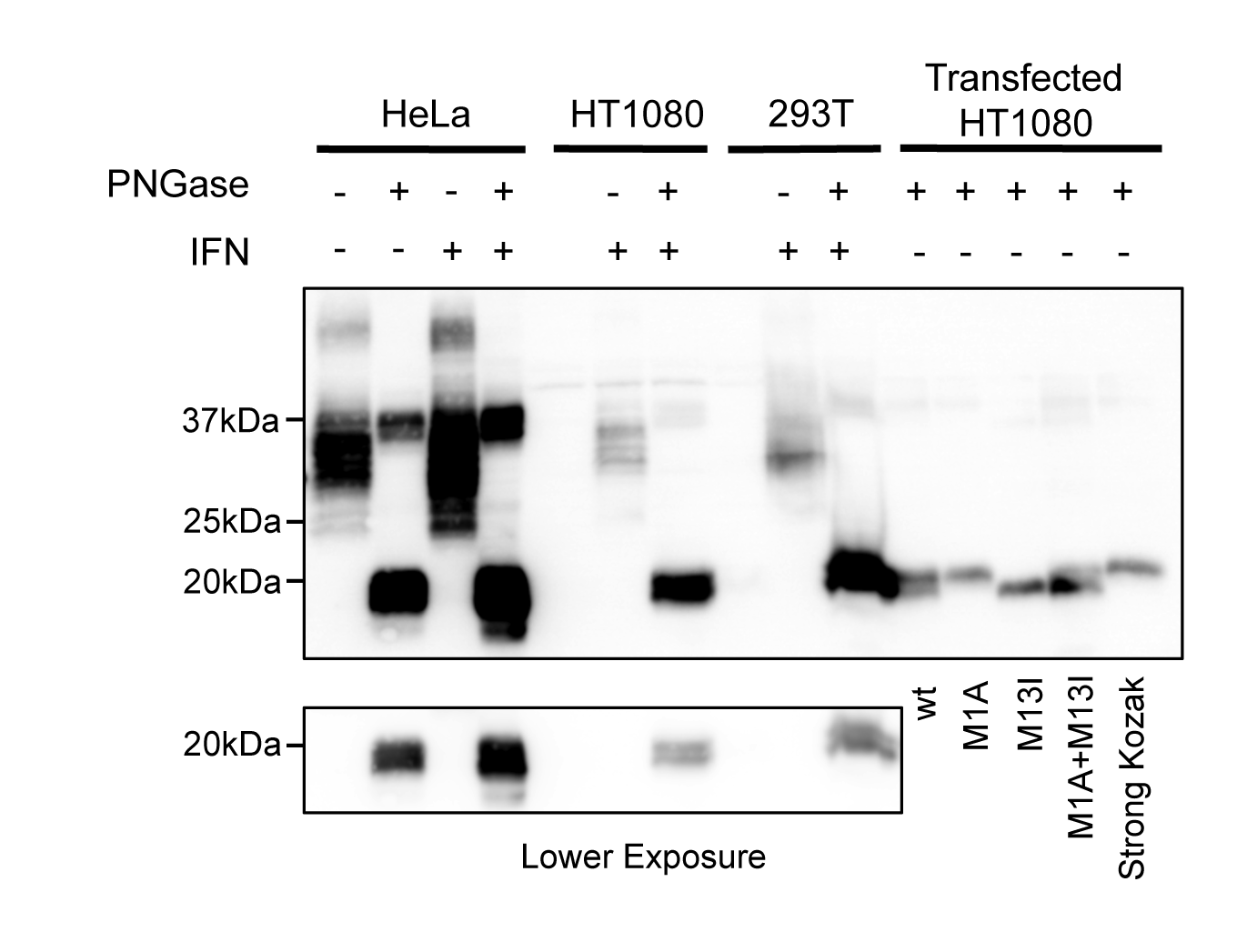

Supplement: Figure S3 — Endogenous expression of Tetherin isoforms. IFN stimulated or unstimulated HeLa, HT1080 and 293T cells were analyzed at 48 h post exposure. Cellular lysates were used directly or digested with PNGase and analyzed by Western blot. A lighter exposure of PNGase treated profile is shown below the main blot. HT1080 cells transiently expressing wt, l-Tetherin, s-Tetherin, l+s-Tetherin or wt Strong Kozak mutants were harvested, PNGase treated and analyzed adjacent to endogenous Tetherin samples. (TIF) [file ppat.1002931.s003.tif]

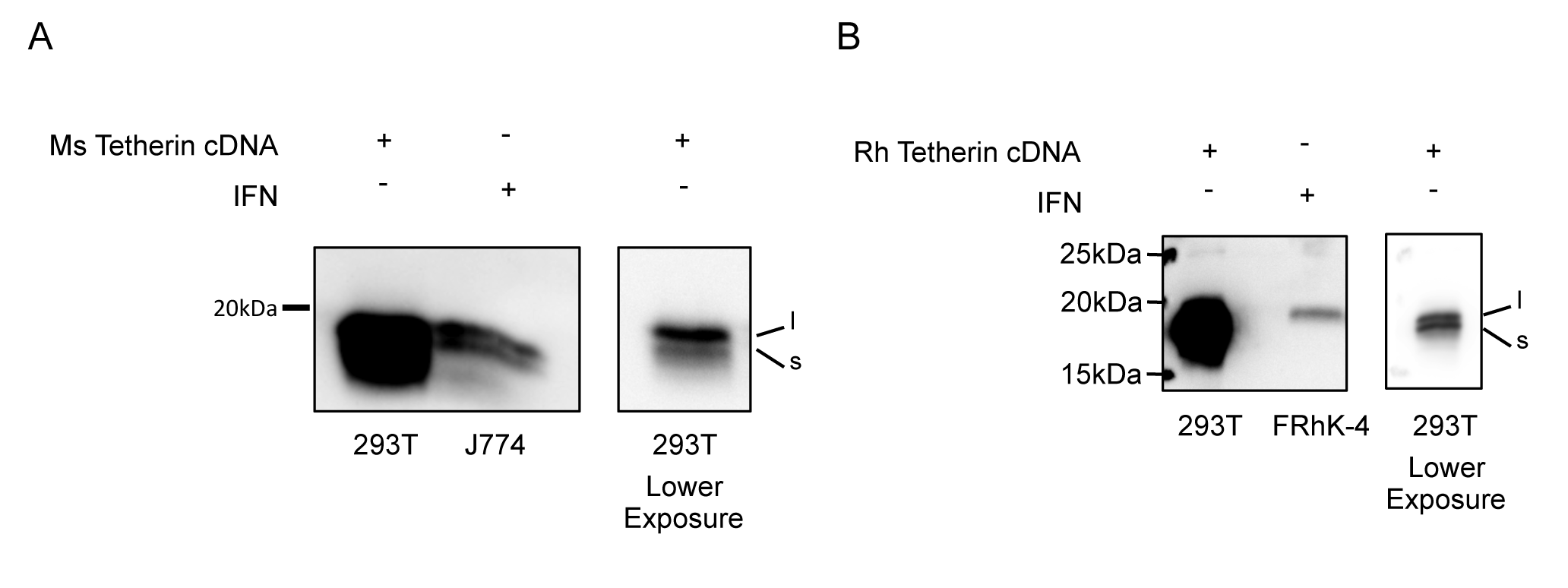

Supplement: Figure S4 — Expression profiles of rhesus and murine Tetherin. (A) 293T cells were transfected with murine Tetherin cDNA. Concurrently, murine J774 cells were IFN treated for 48 h. Lysates were PNGase treated and analyzed by Western blot using an anti-mouse CD317 antibody (BioLegend, 127101). Shorter exposure of the transfected 293T cells is shown to the right. (B) Lysates from 293T cells transiently expressing rhesus Tetherin cDNA were analyzed adjacent to lysates of rhesus FRhK-4 cells. The right panel is a lighter exposure of the 293T lane from the same blot. While the J774 cells, the murine and rhesus Tetherin cDNAs produce two isoforms, only a single species that corresponds to the upper (presumably l-Tetherin) isoform is seen in FRhK-4 cells. (TIF) [file ppat.1002931.s004.tif]

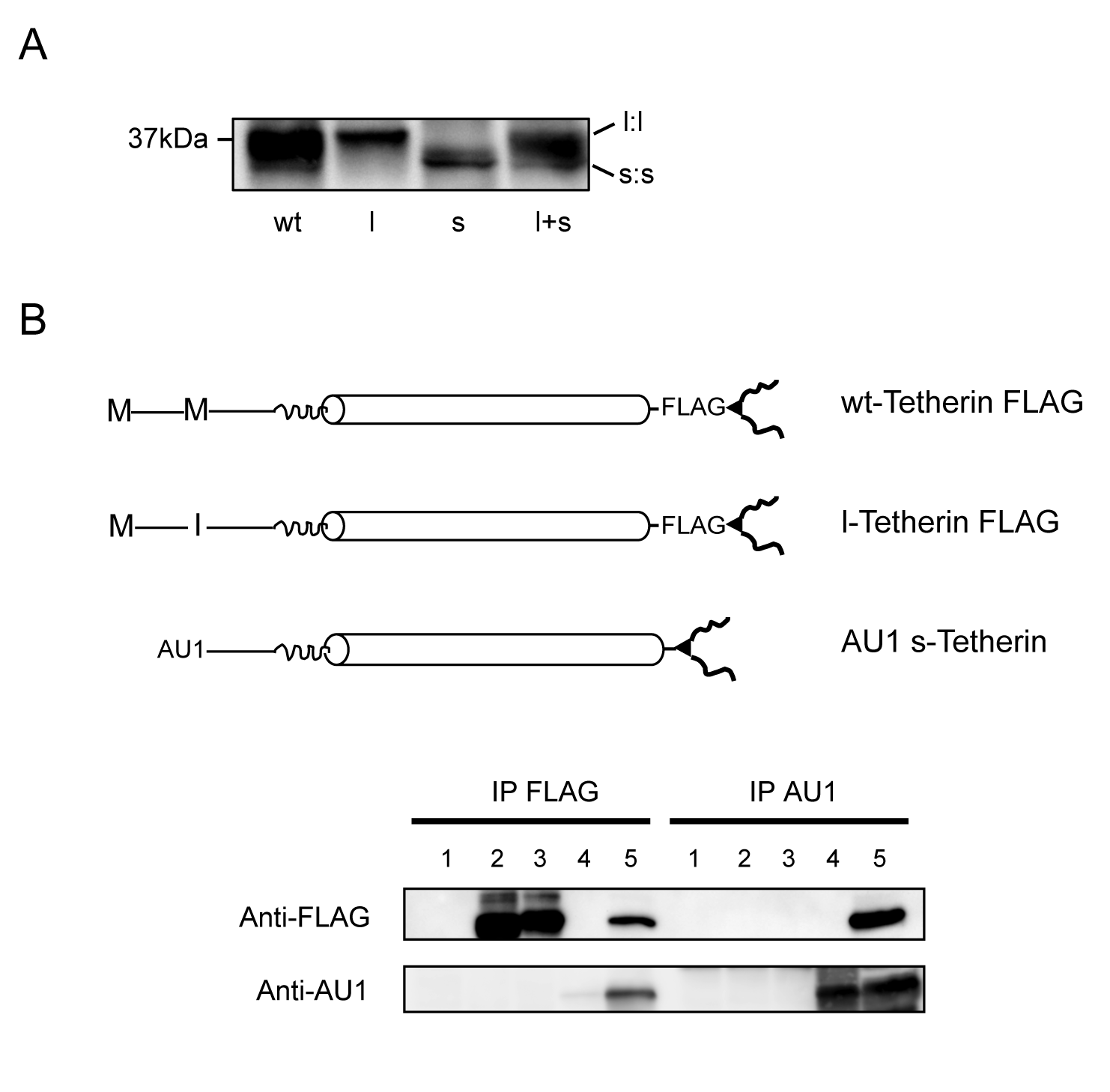

Supplement: Figure S5 — Isoforms produce homo- and heterodimers. (A) HT1080 cells transiently expressing Tetherin mutants form homodimers. HT1080 transfected with either wt, l-, s- or l+s-Tetherin were lysed in RIPA buffer. Lysates were PNGase treated for 2 h without denaturation. Deglycosylated samples were analyzed under non-reducing conditions and probed for Tetherin using anti-BST2 rabbit sera. l:l Long homodimers; s:s short homodimers. (B) Co-immunoprecipitation of epitope tagged isoforms. Cartoon of differentially tagged l- and s-Tetherin expression vectors with tags adjacent to the GPI anchor additions site or at the amino terminus respectively. Epitope tagged Tetherin isoforms were transiently expressed in 293T cells then RIPA lysates were precipitated using the indicated antibodies. Precipitates were analyzed by SDS/PAGE and Western blot. Lane 1, mock transfected; Lane 2, wt-Tetherin FLAG; Lane 3, l-Tetherin FLAG; Lane 4, AU1 s- Tetherin; Lane 5, l-Tetherin FLAG+AU1 s- Tetherin. (TIF) [file ppat.1002931.s005.tif]

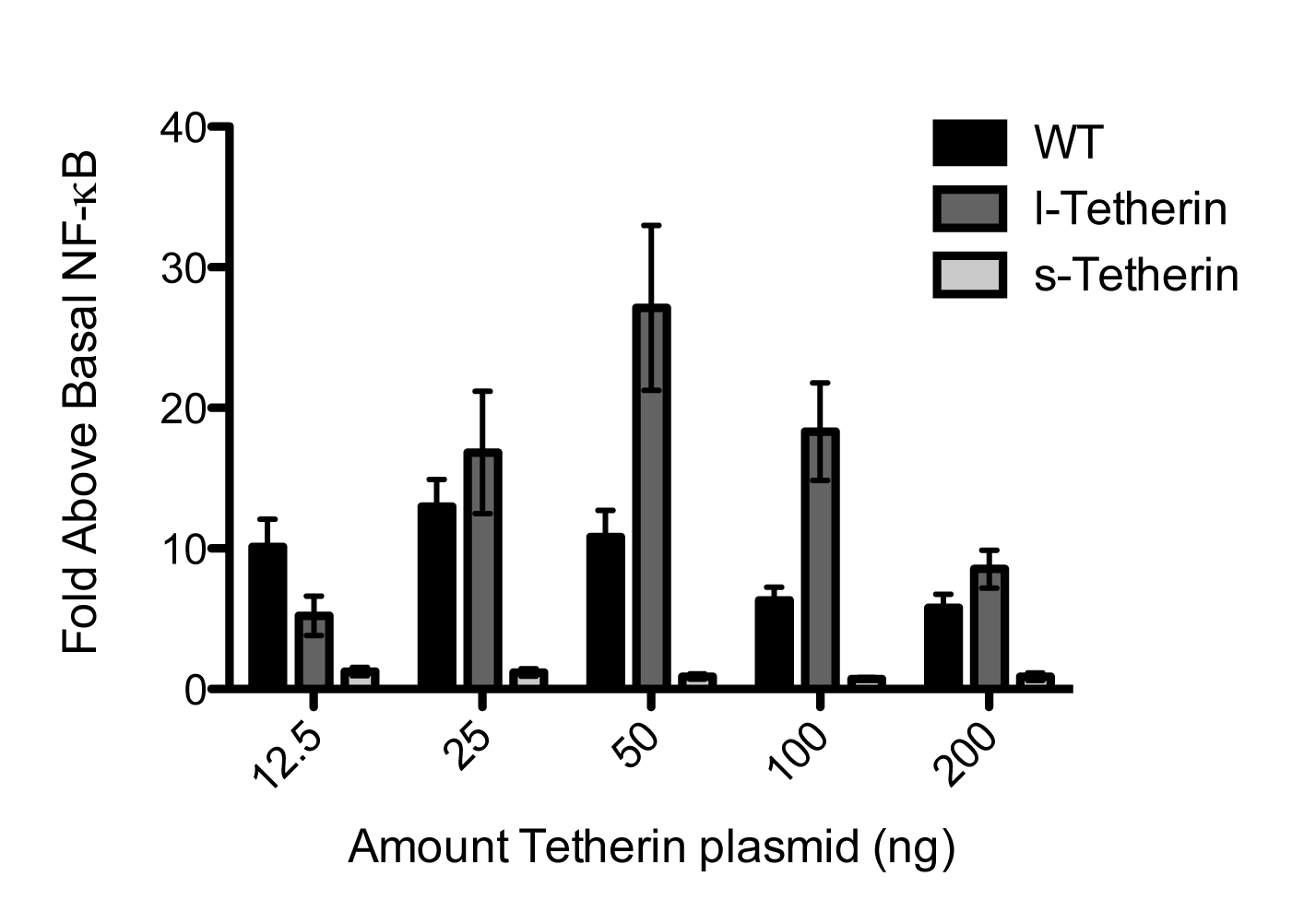

Supplement: Figure S6 — NF-κB induction varies with Tetherin expression level. 293T cells (2×10^5^) were transiently transfected with a range of amounts of Tetherin expression plasmid (12.5, 25, 50, 100 or 200 ng) and a constant amount NF-κB luciferase reporter. Total DNA transfected was kept constant by including empty vector. The results consistently show a bell shaped response for NF-κB activation by Tetherin. From these results 50 ng was chosen as an optimal amount of plasmid for the NF-κB activation assays. This graph is a representative experiment done in triplicate, bars = SD. (TIF) [file ppat.1002931.s006.tif]
