# Supplementary material for: Identification of Alternatively Translated Tetherin Isoforms with Differing Antiviral and Signaling Activities
Source: PLoS Pathog. 2012 Sep 27;8(9):e1002931. doi: 10.1371/journal.ppat.1002931 (PMC3460627; doi:10.1371/journal.ppat.1002931)
Supplement: Text S1 — Supplementary Methods. (DOCX) [file ppat.1002931.s007.docx]

**Supplementary Methods**

**Coimmunoprecipitation**

293T cells were transfected with l-Tetherin FLAG and/or AU1 s-Tetherin. Cells were lysed in RIPA buffer and cleared as described above. Lysates incubated with either anti-FLAG rabbit (Sigma) or anti-AU1 rabbit (Bethyl Lab. Inc.) antibodies bound to Protein A Dynabeads (Invitrogen) for 4 hours. Lysates washed three times using RIPA buffer. Beads were boiled in RIPA+6x SDS/PAGE loading buffer for Western blot analysis. FLAG-tagged l-Tetherin was detected using a FLAG-HRP conjugated antibody (Sigma). s-Tetherin was visualized using the anti-AU1 rabbit antibody described above.
